# A case of refractory immune checkpoint inhibitor‐induced colitis with *Clostridioides difficile* infection

**DOI:** 10.1002/deo2.176

**Published:** 2022-10-17

**Authors:** Yukito Okura, Katsumasa Kobayashi, Yurina Yamada, Makoto Furuya, Naoki Kitano, Eri Oshina, Mana Matsuoka, Takahito Nozaka, Yoshihiro Tashiro, Ayako Sato, Masato Yauchi, Taichi Matsumoto, Yohei Furumoto, Toru Asano, Seishin Azuma

**Affiliations:** ^1^ Department of Gastroenterology Gyotoku General Hospital Chiba Japan; ^2^ Department of Gastroenterology Tokyo Metropolitan Bokutoh Hospital Tokyo Japan

**Keywords:** Clostridioides difficile infection, immune checkpoint inhibitors‐induced colitis, immune‐related adverse events, renal cell carcinoma, ulcerative colitis

## Abstract

The clinical symptoms of an immune checkpoint inhibitor (ICI)‐induced colitis are similar to those of ulcerative colitis. ICI‐induced colitis, like ulcerative colitis, may be complicated by other colitis, such as *Clostridioides difficile* infection (CDI). A 72‐year‐old man was admitted because of watery and bloody stools 10 times a day after three courses of nivolumab (antibodies against programmed death 1) and ipilimumab (cytotoxic T‐lymphocyte‐associated antigen‐4) for stage IV renal cell carcinoma. Colonoscopy revealed erythema and multiple erosions in the colon. Histopathological examination of colonic mucosa revealed diffuse inflammatory cell infiltration and apoptosis. The initial cytomegalovirus antigen test and *C. difficile* detection assay results were negative. Based on these findings, we diagnosed the patient with ICI‐induced colitis and discontinued ICI therapy. The symptoms did not improve despite the administration of Prednisolone and infliximab. A repeat colonoscopy revealed a new appearance of pseudomembranes from the sigmoid colon to the rectum one month after the start of these treatments. At this point, the patient tested positive for *C. difficile*. With treatment with vancomycin for CDI, the abdominal symptoms gradually decreased. Nivolumab alone was cautiously restarted. However, no colitis recurrence and further tumor reduction were observed. Here, we report our experience of a case of refractory ICI‐induced colitis complicated by CDI. ICI‐induced colitis may be complicated by CDI and should be carefully treated with repeated CDI testing if refractory to treatment. We believe that our observation will provide helpful information for determining an appropriate treatment strategy for ICI‐induced colitis.

## INTRODUCTION

In recent years, immune checkpoint inhibitors (ICI) have been widely used for cancer treatment, with their applications expanding to various types of cancer. Antibodies against programmed death 1 (PD‐1) and cytotoxic T‐lymphocyte‐associated antigen‐4 (CTLA‐4) are widely used for cancer treatment. ICI usage is associated with multiple immune‐related adverse events (irAEs), with gastrointestinal disorders such as diarrhea and colitis being one of the most significant adverse events reported in clinical practice. The frequency of diarrhea/colitis is 12.1/0.7% for anti‐PD‐1 agents, 30.2/5.7% for anti‐CTLA‐4 agents, and 45/13% for anti‐PD‐1 agents combined with anti‐CTLA‐4 agents.^1^, ^2^ Generally, ICI‐induced colitis is similar to ulcerative colitis (UC), and therefore, similar treatment strategies are followed for treating both of them. In addition, similar to the clinical course of UC, few studies have reported the resistance to treatment due to complications of *Clostridioides difficile* infection (CDI),^3^, ^4^ which may be severe and fatal at times, and thus, demands appropriate treatment.

Regarding metastatic renal cell carcinoma (RCC), the combination therapy of nivolumab and ipilimumab, both of which are ICI, are effective,^5^ however, few studies have reported colitis related to ICI. Herein, we report an unusual case of refractory ICI‐induced colitis associated with CDI during nivolumab and ipilimumab therapy for RCC.

## CASE REPORT

**FIGURE 1:**
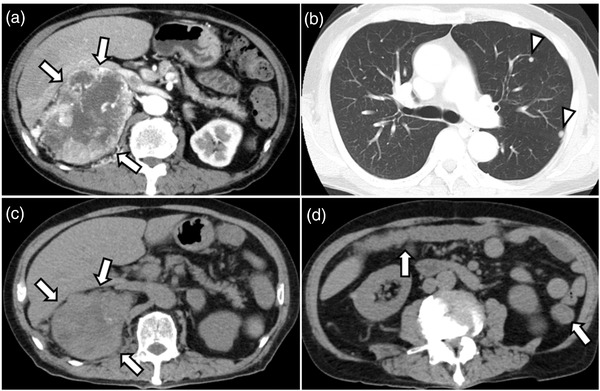
Computed tomography (CT) findings. (a, b) First abdominal CT shows a 15 cm tumor in the right kidney with heterogeneous contrast in the arterial phase (arrows) with multiple lung metastases (arrowheads). (c) After three courses of immune checkpoint inhibitor therapy, CT showed that the primary tumor had decreased in size (arrows). (d) Three days after the end of the third course, CT shows wall thickening throughout the colon (arrows)

**FIGURE 2:**
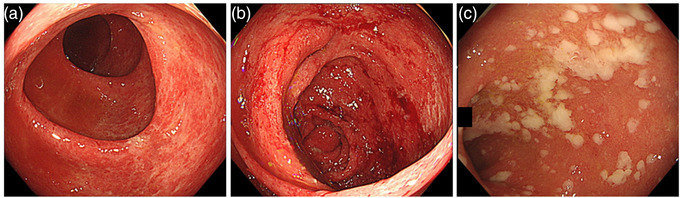
Endoscopic findings. (a, b) Colonoscopy showing erythema, loss of normal vascular pattern, and multiple erosions without ulcers in the ascending and sigmoid colon. (c) Colonoscopy showing pseudomembranes with suspected *Clostridioides difficile* infection in the rectum

**FIGURE 3:**
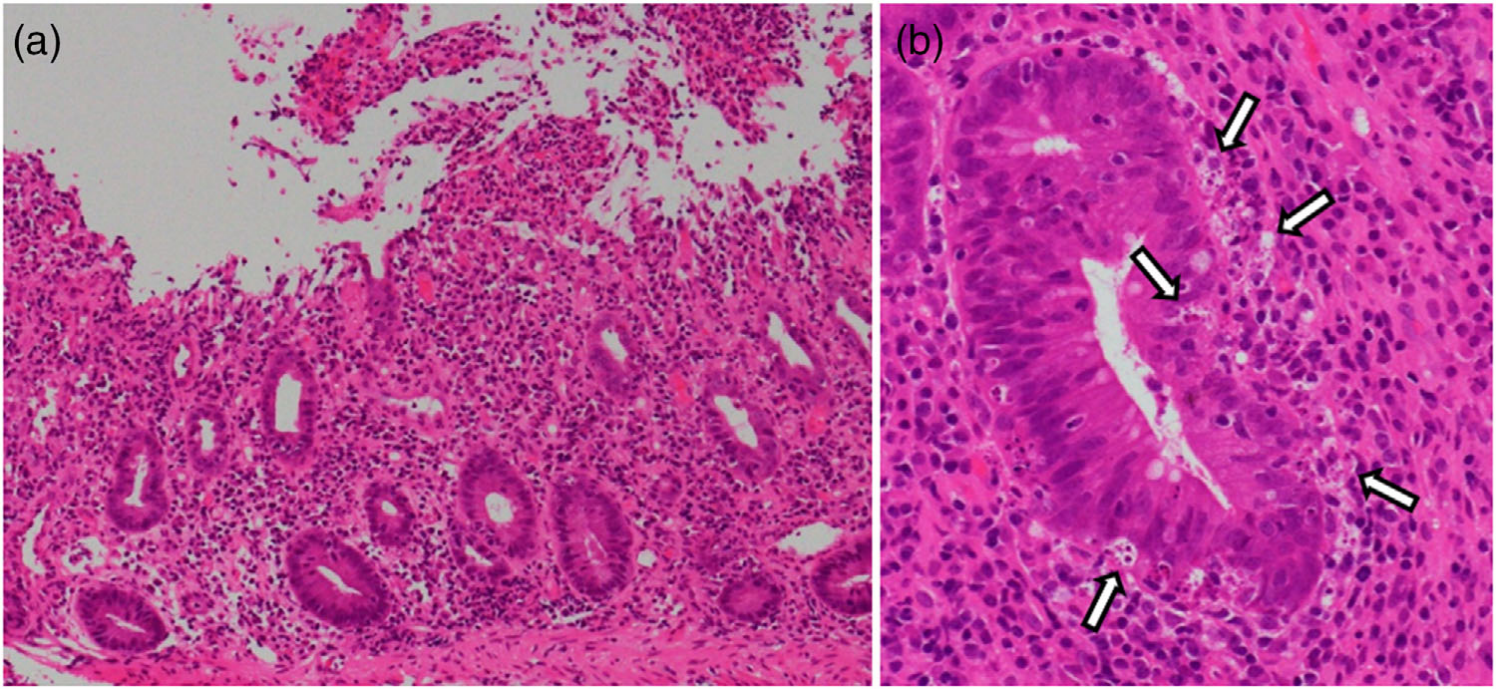
Histopathological findings of hematoxylin and eosin staining. (a) Diffuse inflammatory cell infiltration in the stroma of the intrinsic mucosal layer in the colonic mucosa. (b) Lymphocytic infiltration with apoptosis (arrows)

A 72‐year‐old male with a right kidney mass identified during a medical examination presented to us without any symptoms. Computed tomography (CT) revealed a 15 cm tumor in the right kidney with multiple lung metastases (Figure 1a,b). The patient was diagnosed with RCC clinically staged as cT4N0M1, stage IV (according to the edition of the tumor‐node‐metastasis classification of the International Union Against Cancer)/intermediate risk (according to the International Metastatic RCC Database Consortium Risk Score) based on CT findings. We selected nivolumab plus ipilimumab combination therapy (nivolumab 240 mg/body weight, ipilimumab 1 mg/kg, administration interval 3 weeks) as the first‐line treatment. After three courses of ICI therapy, CT showed a decreased primary tumor and lung metastases (Figure 1c). Three days after the end of the third course, diarrhea appeared and gradually worsened with more than 10 episodes of watery and bloody stools. CT showed wall thickening throughout the colon (Figure 1d). Colonoscopy revealed erythema, loss of normal vascular pattern, and multiple erosions without ulcers throughout the colon (Figure 2a,b). Histopathological findings of the colonic mucosa showed diffuse inflammatory cell infiltration and apoptosis (Figure 3a,b), and no cytomegalovirus (CMV) inclusion bodies were observed. Moreover, stool and *C. difficile* detection assay (*C. difficile* antigen, toxin, and stool culture tests) results were negative. Based on these findings, he was diagnosed with ICI‐induced colitis; therefore, ICI was discontinued, and administration of prednisolone 30 mg/day (0.5 mg/kg) was started without antibiotics.^4^

On the sixth day of steroid initiation, the prednisolone dose was increased to 60 mg/day (1 mg/kg) because of persistent diarrhea (6–8 times a day), despite the disappearance of the bloody stool. The second colonoscopy showed redness, erosion, and ulceration predominantly in the left‐sided colon, suggesting steroid resistance. After confirming negative results for infectious colitis by performing stool assay and *C. difficile* detection assay again, infliximab, and anti‐TNF‐α monoclonal antibodies, (300 mg/body; 5 mg/kg) were administered twice (0 and 2 weeks). Despite two doses of infliximab, diarrhea persisted (4–6 times a day). On the third colonoscopy (30th day after steroid initiation), new pseudomembranes appeared from the sigmoid colon to the rectum (Figure 2c). At this point, the *C. difficile* detection assay was positive, and we diagnosed the CDI complications with ICI‐induced colitis. After treatment with vancomycin 500 mg/day for 10 days, diarrhea disappeared, and abdominal symptoms improved. Prednisolone was gradually reduced to a stop in 8 weeks, with no incidence of symptoms flaring up. Three months later, the ICI‐induced colitis had not recurred for 5 months, despite resuming nivolumab monotherapy.

## DISCUSSION

The clinical symptoms of ICI‐induced colitis are similar to those of UC and hence, are treated similarly. Treatment‐resistant ICI‐induced colitis such as UC may be complicated by other types of colitis, such as CDI or CMV. In this case, CDI complications were negative initially; however, re‐examination upon each treatment failure led to the accurate diagnosis and treatment of ICI‐induced colitis complications. Furthermore, the re‐challenge of ICI administration after irAEs was successful. The clinical course of this case may suggest developing a treatment strategy for ICI‐induced colitis.

Immune checkpoint molecules such as PD‐1 and CTLA‐4 are immunosuppressive receptors that inhibit excessive immune and inflammatory responses. ICI activate tumor immunity by suppressing the activation of cytotoxic T cells by immune checkpoint molecules. ICI cause autoimmune disease‐like symptoms by releasing the autoimmune response that is initially suppressed by immune checkpoint molecules. Therefore, it has been reported that ICI‐induced colitis caused by nivolumab and ipilimumab presents similar symptoms to UC.^6^ Therefore, if symptoms of colitis like UC occur during ICI treatment, ICI‐induced colitis is generally diagnosed by endoscopy, necessitating rapid endoscopy for proper diagnosis of ICI‐induced colitis.

Although it is common for CDI to develop after antibiotics, this case did not receive such treatments. In contrast, factors other than antibiotics have been reported to be risk factors for CDI, such as advanced age, hospitalization, proton pump inhibitors, chemotherapy, and inflammatory bowel disease (IBD), including UC.^7^ IBD could be a risk factor for CDI due to decreased diversity of gut microbiota and immune disorders.^8^ Moreover, ICI‐induced colitis is likely to be a risk factor for the development of CDI because it has a clinical and pathological course similar to that of UC. Additionally, long‐term administration of prednisolone and anti‐TNF‐α agents has been suggested to be a risk factor for CDI, and treatment for ICI‐induced colitis may cause CDI.^7^, ^9^ We speculate that the overlap of ICI treatment and administration of prednisolone and anti‐TNF‐α agents led to complications of CDI during treatment, resulting in patients turning resistant to therapy. However, the initial symptoms were caused by ICI‐induced colitis alone. If the treatment of ICI‐induced colitis is unsuccessful, as in this case, or if the clinical course is different from what was expected, a further examination should be considered. Even if CDI is not diagnosed at the initial examination, repeat examinations (colonoscopy, stool, etc.) can lead to the correct diagnosis of CDI complications, facilitating appropriate treatment.

There is only limited data on the safety of the re‐challenge of ICI after the development of ICI‐induced colitis. The American Society of Clinical Oncology guidelines recommends that ICI should not be resumed in grade 4 patients with ICI‐induced colitis and that anti‐CTLA‐4 agents should be permanently discontinued in grade 3 patients. In grade 3 colitis, anti‐PD‐1 agents can be considered for re‐challenge after recovery to grade 1.^4^ In one study, the pooled incidence of all‐grade and high‐grade irAEs after re‐challenge in patients with cancer was 34.2%, and 11.7%, respectively.^10^ In contrast, anti‐PD‐1 agents rechallenge, compared to anti‐CTLA‐4 agents, has been reported to be associated with a lower all‐grade irAE recurrence.^10^ In this case, anti‐PD‐1 alone agents were cautiously restarted due to a significant reduction in tumor growth due to the prior treatment. Fortunately, there was no recurrence of colitis, and further tumor reduction was observed. However, there is a possibility of recurrence of ICI‐induced colitis in the long term, and further careful observation is required.

In summary, we report a refractory case of ICI‐induced colitis complicated by CDI. Patients with treatment‐resistant ICI‐induced colitis should be treated cautiously to avoid CDI complications during treatment. We believe this case will provide helpful information for determining an appropriate treatment strategy for ICI‐induced colitis.

## CONFLICT OF INTEREST

None.

## FUNDING INFORMATION

None.

## ETHICS STATEMENT

All procedures were performed according to the principles of the Declaration of Helsinki and its later amendments.
